# Transcriptomic detection of *Candidatus* Allocryptoplasma (Anaplasmataceae) in Galápagos marine iguanas (*Amblyrhynchus cristatus*, Iguanidae)

**DOI:** 10.1186/s13071-025-07138-7

**Published:** 2025-11-28

**Authors:** Flora Uesseler, Lena Werner, Stefan Schaffer, Alejandro Ibáñez, Scott Glaberman, Diego Páez-Rosas, Juan M. Guayasamin, Michael Hofreiter, Sebastian Steinfartz, Franziska Anni Franke-Gerth

**Affiliations:** 1 Evolutionary Adaptive Genomics, Institute for Biochemistry and Biology, University of Potsdam, Potsdam, Germany; 2 Molecular Evolution and Animal Systematics, Institute of Biology, University of Leipzig, Leipzig, Germany; 3 Faculty of Biology and Environmental Protection, Department of Ecology and Vertebrate Zoology, University of Lodz, Lodz, Poland; 4 Centre for Environmental Research and Justice, School of Biosciences, University of Birmingham, Birmingham, UK; 5 Galápagos Science Center, Universidad San Francisco de Quito USFQ and University of North Carolina Chapel Hill, San Cristóbal, Galápagos Ecuador; 6 Unidad Técnica Operativa San Cristóbal, Dirección del Parque Nacional Galápagos, San Cristóbal, Galápagos Ecuador; 7 Laboratorio de Biología Evolutiva, Colegio de Ciencias Biológicas y Ambientales COCIBA, Instituto Biósfera, Universidad San Francisco de Quito USFQ, Campus Santiago Gangotena, Quito, Ecuador

**Keywords:** Ticks, Vector-borne diseases, 16S ribosomal RNA

## Abstract

**Background:**

Globally, the disease ecology of reptiles remains understudied, even for threatened and iconic species such as the Galápagos marine iguana (*Amblyrhynchus cristatus*). Although marine iguanas are parasitized by distinct species of ticks and mites, research on vector-borne diseases for this species is limited.

**Methods:**

In this study, we detected 16S ribosomal RNA (rRNA) sequences of *Candidatus* Allocryptoplasma in transcriptomic data from marine iguana blood samples. These 16S rRNA sequences were further characterized through phylogenetic analysis and a haplotype network.

**Results:**

Our analysis revealed the first molecular evidence for the infection of marine iguanas with *Candidatus* Allocryptoplasma, a candidate genus in the family Anaplasmataceae with unknown pathogenic potential, likely transmitted by ticks. Phylogenetic analysis of the novel 16S rRNA sequences together with available Anaplasmataceae sequences confirmed their assignment to this candidate genus. A haplotype network analysis indicated that the agent infecting the marine iguana represents a distinct lineage within the known *Ca*. Allocryptoplasma diversity.

**Conclusions:**

*Candidatus* Allocryptoplasma had a high prevalence within marine iguanas, infecting individuals across most of the geographical range of this species. To elucidate the transmission dynamics of this bacterium in the Galápagos ecosystem, ectoparasites of the marine iguana and shared vertebrate hosts should be screened for infection with *Ca*. Allocryptoplasma.

**Graphical Abstract:**

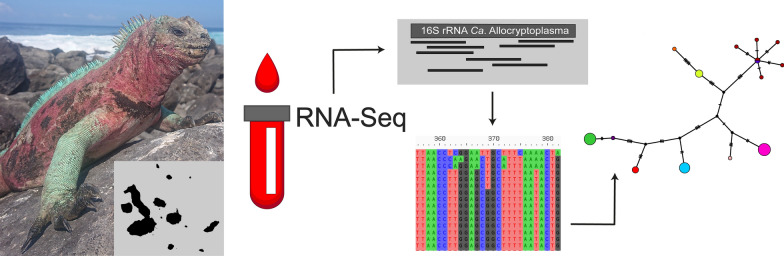

**Supplementary Information:**

The online version contains supplementary material available at 10.1186/s13071-025-07138-7.

## Background

Reptiles are hosts to a diverse range of bacteria, fungi, viruses, and macroparasites, the latter including myxosporea, entamoebae, coccidia, trypanosomes, hemogregarines, as well as helminths such as nematodes, trematodes, and cestodes [1, 2]. Although pathogen diversity has been studied in some taxa, such as freshwater turtles [3], research on the epidemiology and ecology of reptile diseases remains limited [4], particularly in Latin America, a region rich in reptile biodiversity [5].

Parasitism and disease can adversely affect host physiology, influencing fitness measures such as offspring phenotype, reproductive success, physiological performance, and survival [1]. These impacts can lead to changes in thermoregulatory [6] and social behavior [7], and interspecific competition [8, 9]. Because parasites influence host physiology and behavior, understanding their diversity and ecological impacts is important for reptile conservation management, especially, since globally, at least 21.1% of reptile species are classified as threatened [10].

The role of tick-borne diseases and their zoonotic potential has received growing research interest [11–13]. Parasitic mites such as ticks (Ixodida) and some members of the orders Trombidiformes and Mesostigmata are among the main vectors of bacterial pathogens in reptiles [12], transmitting species in the genera *Aeromonas*, *Coxiella*, *Rickettsia*, *Borrelia*, and also *Ehrlichia* and *Anaplasma*, which are members of the Anaplasmataceae family [11]. The Anaplasmataceae family consists of obligate intracellular α-proteobacteria, which replicate within vacuoles of eukaryotic host cells [14]. It includes four established genera, *Wolbachia*, *Neorickettsia*, *Ehrlichia*, and *Anaplasma* [15]. Owing to the difficulty of isolating and culturing obligate intracellular bacteria in the laboratory, four other taxa within the Anaplasmataceae currently have the status of putative genera: *Candidatus* Neoehrlichia [16], *Candidatus* Xenohaliotis [17], *Candidatus* Xenolissoclinum [18], and *Candidatus* Allocryptoplasma [19].

The putative genus *Ca*. Allocryptoplasma was first detected in ticks (*Ixodes pacificus*) from California and initially described as *Candidatus* Cryptoplasma [19], later corrected to *Ca*. Allocryptoplasma [20]. Since then, genetic evidence for infection with this candidate genus has accumulated in studies on ticks worldwide, including Asia, Europe, Africa, and North and South America [13, 19, 21–23]. *Candidatus* Allocryptoplasma has also been identified in various vertebrate hosts, such as field mice (*Apodemus agrarius*) [24], several lacertid lizard species in Europe [13, 21], and multiple bird species from the Brazilian Pantanal wetland [25]. Furthermore, Ouass et al. [26] demonstrated that *Ca*. Allocryptoplasma isolated from a broad range of host species and geographic locations form a single monophyletic group, placed as the sister lineage to *Anaplasma*. The genetic diversity within *Ca*. Allocryptoplasma is comparable to that of *Anaplasma* and *Ehrlichia*, supporting its status as a candidate genus [26]. The concurrent detection of *Ca*. Allocryptoplasma in lizards and their ectoparasites suggests that ticks and mites may act as vectors, transmitting the bacterium to other vertebrate hosts [13, 21].

The Galápagos marine iguana (*Amblyrhynchus cristatus*) is endemic to the Galápagos archipelago, occurring on the coasts of all major and most minor islands [27]. It is currently classified as vulnerable, with a declining population trend by the International Union for the Conservation of Nature (IUCN) Red List of Threatened Species [28]. Climate change and other anthropogenic disturbances continue to threaten this species and the broader Galápagos ecosystem [29, 30]. Marine iguanas are parasitized by four endemic tick species in the genera *Amblyomma* and *Ornithodoros* [31]. Both marine iguanas [32] and their associated ticks have been found to carry apicomplexan hemoparasites from the genera *Hepatozoon* or *Hemolivia*, or both [33]. Hemogregarines assigned to *Hepatozoon* have also been detected in the mosquito *Aedes taeniorhynchus*, which feeds on the blood of marine iguanas [34]. Beyond these reports, however, vector-borne diseases in marine iguanas remain poorly studied.

Particularly in transcriptomic data, abundant cellular ribosomal RNA (rRNA) sequences often appear as a byproduct, even when methods such as poly(A)-enrichment or rRNA depletion are applied before sequencing [35–37]. Because extensive rRNA reference data are available, this information can be leveraged for characterizing nonhost sequences. In this study, we utilized such an approach to analyze 16S rRNA gene sequences from the blood of marine iguanas to investigate the potential presence of the blood-borne bacterium *Ca*. Allocryptoplasma. This revealed infections with *Ca*. Allocryptoplasma in marine iguanas across all sampling sites, comprising most of their geographic distribution. Our results provide the first molecular evidence of *Ca*. Allocryptoplasma infection in marine iguanas, and the first report of this potential emerging pathogen on the Galápagos archipelago. Despite extensive research on marine iguanas, our study underscores the need for further investigation into their disease ecology and more broadly into parasite ecology and epidemiology in reptiles.

## Methods

### Sampling

Blood samples of 56 marine iguanas were collected from 2015 to 2016 from nine different islands of the Galápagos archipelago in Ecuador and preserved in RNAlater (Thermo Fisher Scientific). Sampling locations included the islands of San Cristóbal, Santiago, Pinta, Marchena, Genovesa, Isabela, Fernandina, Española, and Floreana (Table 1).

**Table 1:** Number of sequenced marine iguanas, according to sex and sampling location

Island (abbreviation)	Longitude	Latitude	Number of individuals	Sex composition (male/female)
Española (ESP)	−89.6204	−1.39483	6	3/3
Fernandina (FDA)	−91.38947	−0.44264	7	5/2
Floreana (FL)	−90.50911	−1.31968	6	4/2
Genovesa (GEN)	−89.97349	0.31065	6	6/0
Isabela (IS)	−91.42524	−0.78587	6	6/0
Marchena (MAR)	−90.50774	0.30051	6	4/2
Pinta (PIN)	−90.73948	0.5434	6	3/3
Santiago (SAN)	−90.86495	−0.24215	7	5/2
San Cristóbal (SRL)	−89.62125	−0.92214	6	3/3

### RNA extraction and library preparation

Total RNA extraction from each blood sample was carried out twice using the TRIzol method. Samples were thawed on ice and vortexed briefly, before transferring 500 µl of blood-RNAlater mixture to a fresh tube. After centrifugation for 2 min at 8000*g* at 4 °C, the supernatant was discarded, and 1.5 ml TRIzol (Invitrogen) was added to the pellet, and vortexed briefly. Afterward, the samples were frozen at −80 °C overnight and then thawed on ice the next day. Subsequently, 300 µl of chloroform was added and vortexed for 20 s. The following centrifugation at 10,000*g* for 10 min at 4 °C separated two phases, with the RNA contained in the upper, aqueous phase. The aqueous phase was transferred to a fresh tube, and 750 µl isopropanol was added and mixed by inverting the tube several times. After 10 min of incubation at room temperature, tubes were centrifuged at 10,000*g* for 10 min at 4 °C. The supernatant was discarded and isopropanol precipitation repeated. The pellets were washed with 750 µl 70% ethanol, and another centrifugation step at 5000*g* for 10 min at 4 °C followed. The supernatant was discarded, and the pellet was air-dried for 5 min, before redissolving the pellet in 60 µl RNAse-free water. RNA extracts originating from the same blood sample were pooled.

For the DNAse I treatment, 10 µl AMBION 10 × DNAse I buffer (Invitrogen) and 0.25 µl AMBION RNAse-free DNAse I (2 U / µl) (Invitrogen) were added to 100 µl of RNA extract and then incubated at 37 °C for 30 min. RNA purification was conducted with the RNeasy MinElute Cleanup Kit (QIAGEN, Hilden Germany). RNA concentration and integrity were measured with the Bioanalyzer Eukaryote Total RNA Nano assay using the Agilent RNA 6000 Nano Kit on an Agilent 2100 Bioanalyzer instrument (Agilent Technologies).

Isolation of messenger RNA (mRNA) from 30 to 200 ng total RNA by poly-dT enrichment was performed using the NEBNext Poly(A) mRNA Magnetic Isolation Module (NEB), according to the manufacturer’s instructions. Samples were then directly subjected to the workflow for strand-specific RNA sequencing (RNA-Seq) library preparation (Ultra II Directional RNA Library Prep, NEB) using the NEB Next Adapter from the NEB Next Multiplex Oligos for Illumina Kit, at a final concentration of 0.016 µM. After ligation, unused adapters were depleted by an XP bead (Beckman Coulter) purification step, using a bead-to-sample ratio of 0.9:1. Library amplification and indexing were carried out with a Unique Dual Index strategy, using 12 polymerase chain reaction (PCR) amplification cycles and amplification primers carrying the same sequence for the i7 and i5 indexes (i5: AAT GAT ACG GCG ACC ACC GAG ATC TAC AC NNNNNNNN ACA TCT TTC CCT ACA CGA CGC TCT TCC GAT CT, i7: CAA GCA GAA GAC GGC ATA CGA GAT NNNNNNNN GTG ACT GGA GTT CAG ACG TGT GCT CTT CCG ATC T). The final libraries were again bead-purified (0.9:1) and quantified using a fragment analyzer (Agilent). Sequencing was performed on an Illumina NovaSeq 6000 in 150-base pair (bp) paired-end mode.

### Sequence processing and analysis

Raw reads were trimmed for adapters, potential PhiX contamination, and homopolymers using the program BBduk from the software package BBTools (version 38.96) [38]. The quality of reads was assessed before and after trimming using the FastQC software [39].

SortMeRNA (version 4.3.4) [40] was run to separate rRNA reads from other RNA reads, using the 5S and 5.8S rRNA [41] and the bacterial, archaeal, and eukaryotic SILVA databases [42]. Taxonomic assignment was applied to the rRNA reads using the classify command of the RDP Classifier tool from the RDPTools package (version 2.11) [43]. Only read assignments with a confidence value > 0.9 at genus level were considered further, revealing the presence of rRNA reads belonging to the Anaplasmataceae family among them. The reads assigned to Anaplasmataceae were then further identified as showing the highest sequence similarity to *Ca*. Allocryptoplasma with a search against the National Center for Biotechnology Information (NCBI) nonredundant nucleotide collection database [44] using MegaBLAST [45].

The trimmed rRNA reads were mapped to the 16S rRNA gene of *Ca*. Allocryptoplasma sp. Clone LV17 (GenBank accession no. MG924904.1) with the software HISAT2 (version 2.2.1) [46], using the parameter –rna-strandness RF to account for the directional library type. Duplicates were removed using Picard Tools MarkDuplicates (version 2.25.3) (https://broadinstitute.github.io/picard/), with the parameters REMOVE_DUPLICATES = TRUE and TAGGING_POLICY = All.

We also attempted mapping the trimmed reads of sample ESP16-241, which was among the samples with the highest number of mapped reads for the 16S rRNA gene alignments, to other commonly used marker genes of *Ca*. Allocryptoplasma. The procedure was the same as described above for the 16S rRNA gene, using the following sequences as a reference: *gltA* (GenBank accession no. OQ724618.1), *groEL* (GenBank accession no. OQ724586.1), *rpoB* (GenBank accession no. OQ724570.1), and *sucA* (GenBank accession no. OQ724550.1). No reads mapped for any of these markers, likely owing to the much lower copy number of mRNA molecules transcribed from these genes compared with the more abundant rRNA molecules, as is typical for prokaryotes [47].

While all except one sample contained reads that mapped to the *Ca*. Allocryptoplasma 16S rRNA gene sequence, the mapping rate was low and highly variable between samples (Additional File 1: Supplementary Table S1 and Fig. S1). To construct consensus sequences of the 16S rRNA gene, the alignments of individual libraries were merged according to sampling location, using the merge option of SAMtools (version 10.2.0) [48]. We then generated a consensus sequence from the merged 16S rRNA alignments for each sampling location using the software Geneious Prime (version 2023.2.1) (Biomatters Ltd. Auckland, New Zealand) [49], with a 75% majority rule consensus threshold. Positions with a coverage below 2 were masked as N. As very few reads mapped for all the individual libraries from Isabela Island (Additional File 1: Supplementary Table S1), no consensus sequence could be constructed, and therefore, this sampling location was excluded from downstream analyses. The assignment of the consensus sequences to the genus *Ca*. Allocryptoplasma was verified using a Basic Local Alignment Search Tool (BLAST) search against the nonredundant nucleotide collection database of NCBI [44].

To identify additional publicly available sequences that show high nucleotide similarity with *Ca*. Allocryptoplasma that could be included in the phylogenetic analysis, previous literature of the candidate genus was reviewed and high-confidence hits (*E* = 0.0; percent identity > 98%) from the BLAST search described above were selected. For phylogenetic analysis, we added these publicly available 16S rRNA gene sequences for *Ca*. Allocryptoplasma (Additional file 1: Supplementary Table S2), and other representatives of the Anaplasmataceae family, as well as the outgroup *Rickettsia parkeri* (Additional File 1: Supplementary Table S3) from GenBank to the sequences obtained in our study.

From this dataset, we constructed a multiple sequence alignment (MSA) in AliView (version 1.28) [50] using the MUSCLE algorithm (version 3.8.425) [51]. ModelFinder [52] was applied to choose the best-fit substitution model according to the Bayesian information criterion, which was determined to be the TPM3 + I + G4 model. The MSA of 16S rRNA gene sequences contained 1554 sites, including 381 parsimony-informative sites, providing sufficient resolution for phylogenetic analysis. We inferred a phylogenetic tree from the MSA using the maximum likelihood (ML) method implemented in IQTree 2 (version 2.2.2.7) [53] using 1000 ultrafast bootstrap (UFboot) [54] replicates and the Shimodaira–Hasegawa-like approximate likelihood ratio test (SH-aLRT) [55] with 1000 bootstrap replicates.

In addition, we constructed another MSA as described above, but only including the *Ca*. Allocryptoplasma 16S rRNA sequences. This MSA had a length of 1141 nucleotides and was used to generate a median-joining haplotype network in PopArt (version 1.7) [56].

Plots were generated in RStudio version 2025.05.1 + 513 [57] using the Tidyverse suite of packages [58]. Raw rRNA reads for each individual library were deposited in the European Nucleotide Archive (ENA) under the BioProject no. PRJEB101020.

## Results

We detected the presence of *Ca*. Allocryptoplasma in marine iguana blood through the identification of 16S rRNA sequences from transcriptomic data. The taxonomic identity of these sequences was confirmed by aligning reads to a reference *Ca*. Allocryptoplasma 16S rRNA gene and constructing a ML phylogeny with other members of the Anaplasmataceae family. All analyzed samples in this study contained reads that mapped to the *Ca*. Allocryptoplasma reference, except for one from Marchena (Additional File 1: Supplementary Table S1). The number of normalized mapped reads was highly variable, with samples from Isabela Island consistently showing low numbers (Additional File 1: Supplementary Table S1 and Fig. S1). The highest number of normalized mapped reads was observed in individuals sampled from the Islands of Pinta and Española, although these locations also showed high interindividual variability (Additional File 1: Supplementary Table S1 and Fig. S1).

Consensus 16S rRNA sequences of *Ca*. Allocryptoplasma, constructed from merged alignments of each population, showed one to three ambiguous nucleotides: one for Floreana, Genovesa, Marchena, and Santiago; two for Fernandina, Pinta, and San Cristóbal; and three for Española. Visual inspection confirmed that these ambiguous positions were also present in individual sample alignments as well as in the merged alignments. The presence of ambiguous nucleotides could indicate coinfection with multiple strains of *Ca*. Allocryptoplasma within individual marine iguanas, characterized by sequence heterogeneity.

**Fig. 1:**
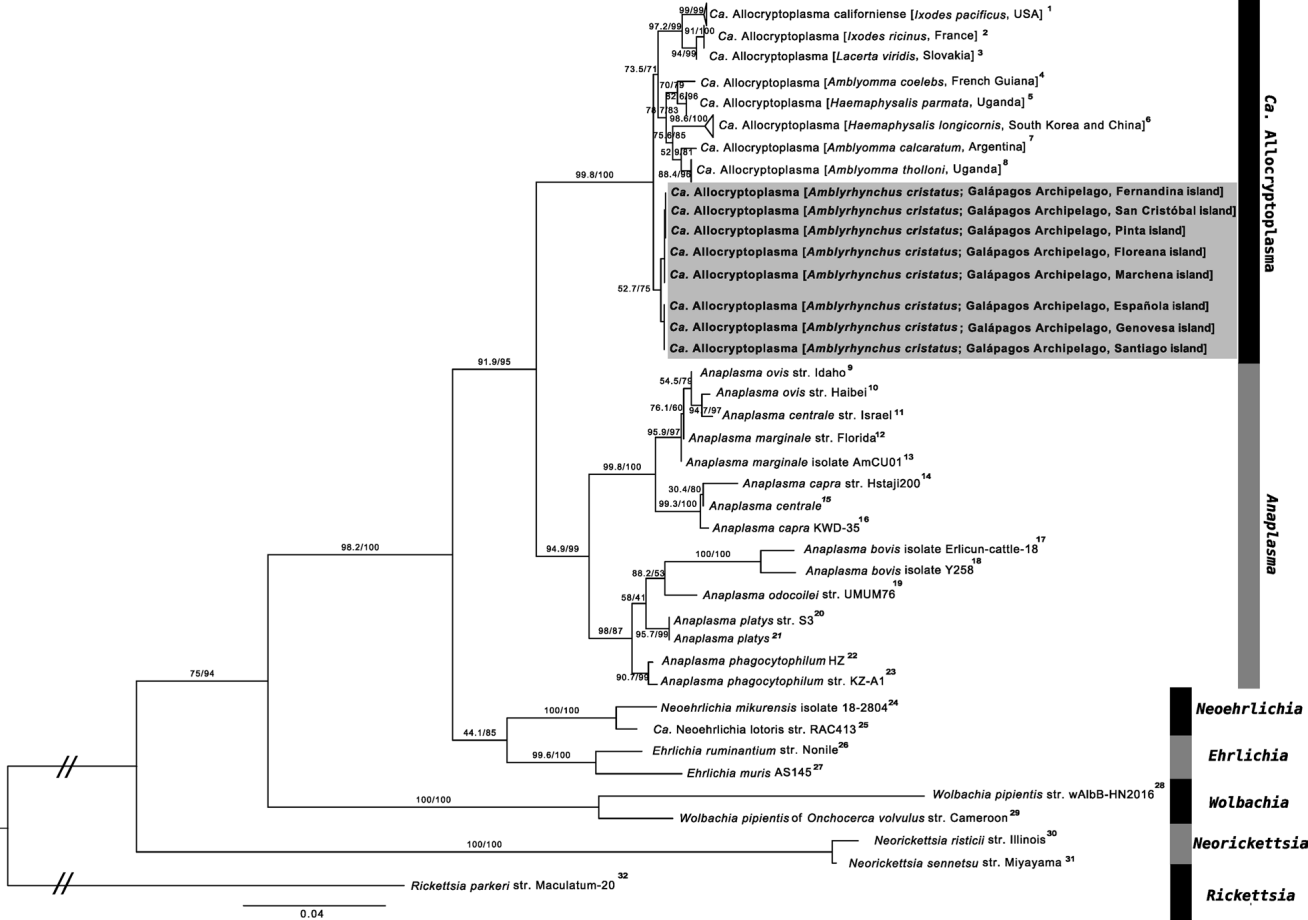
Maximum likelihood phylogeny of 16S ribosomal RNA gene sequences (1554 bp) of *Candidatus* Allocryptoplasma and selected representatives of the family Anaplasmataceae, with *Rickettsia parkeri* as outgroup. Sequences newly obtained from this study are marked in bold and within the gray box. Superscript numbers in the tree tip labels indicate the corresponding GenBank accession numbers, which are listed in Additional File 1: Supplementary Table S2 and Additional File 1: Supplementary Table S3. The best-fit substitution model was determined to be TPM3 + I + G4. The scale bar represents substitutions per site. Support values at the nodes correspond to SH-aLRT and UFboot values with 1000 replicates. Branches that were shortened for visualization purposes are marked by a break in the branch with double dashes (//)

In the ML phylogeny, all *Ca*. Allocryptoplasma 16S rRNA sequences formed a well-supported monophyletic clade, including those isolated from marine iguana blood samples in this study (Fig. 1). A sister relationship between *Ca*. Allocryptoplasma and the genus *Anaplasma* was also supported. More distantly related representatives of the Anaplasmataceae formed monophyletic clades, except for two sequences of *Anaplasma centrale*, which were polyphyletic: one clustered with *Anaplasma ovis* and the other with *Anaplasma capra*. The newly sequenced *Ca*. Allocryptoplasma sequences from marine iguanas formed the most basal split within the genus, separating them from all other *Ca*. Allocryptoplasma sequences, but this position is weakly supported (SH-aLRT, 52.7 < 80; UFboot, 75 < 95).

**Fig. 2:**
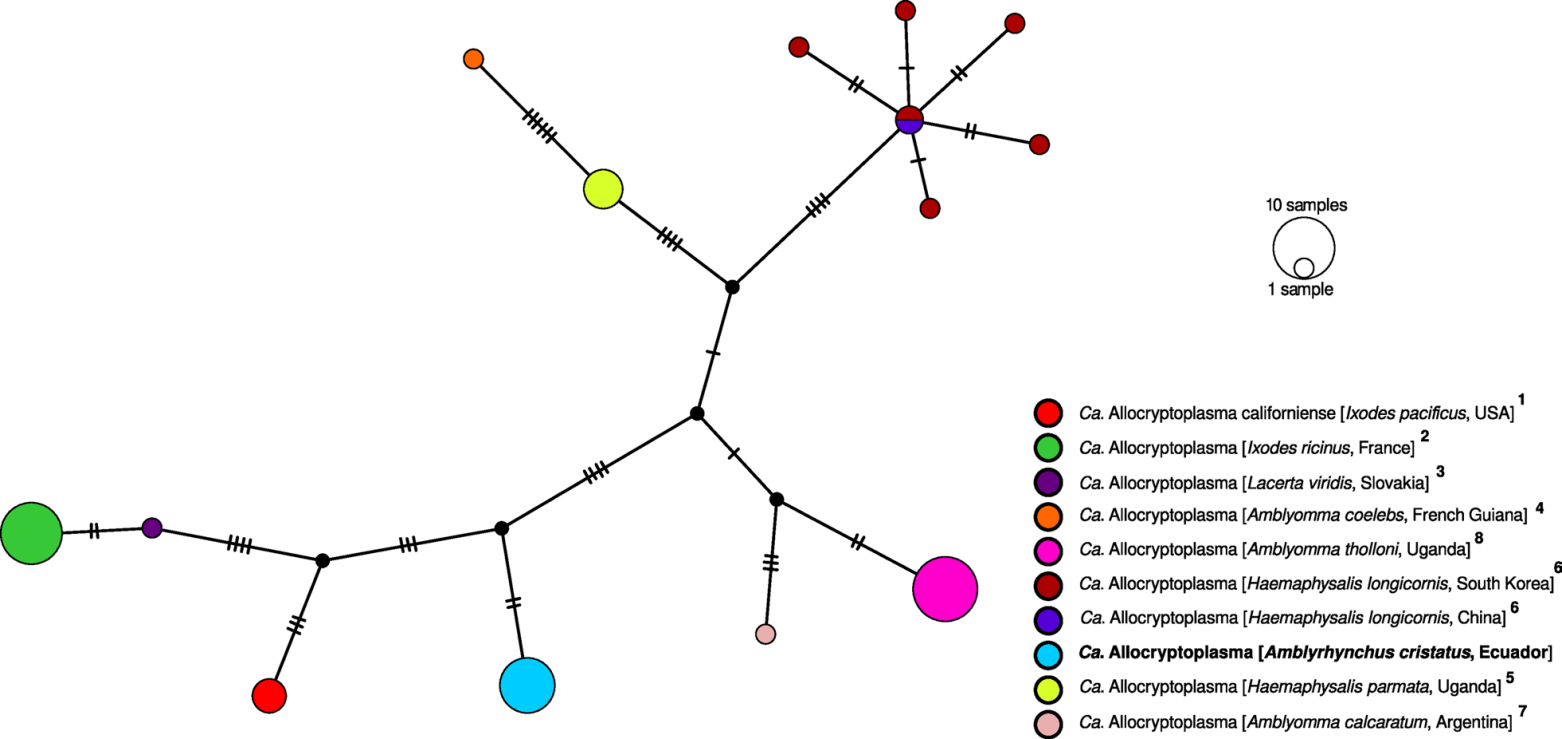
Haplotype network of 16S ribosomal RNA gene sequences of *Candidatus* Allocryptoplasma (1141 bp), using the median-joining algorithm. Colored circles indicate distinct haplotypes, small black circles represent undetected haplotypes, and crossbars represent single mutational steps. Each color represents a combination of host species and country of origin, and the associated superscript numbers in the legend indicate the corresponding GenBank accession numbers that are listed in Additional File 1: Supplementary Table S2. Sequences newly obtained from this study are indicated in bold

The corresponding 16S rRNA haplotype network assigned 14 different haplotypes on the basis of 37 segregating sites and 26 parsimony-informative characters (Fig. 2). The *Ca*. Allocryptoplasma sequences isolated from marine iguanas represented a unique haplotype, distinct from the other described strains. The marine iguana haplotype was most similar to *Ca*. Allocryptoplasma californiense from *I. pacificus* ticks in California, separated by 11 mutational steps in the haplotype network.

## Discussion

In this study, we provide novel molecular evidence for the occurrence of *Ca*. Allocryptoplasma on the Galápagos Islands, and more specifically, for the infection of marine iguanas with this candidate genus of bacteria. As recent studies on *Ca*. Allocryptoplasma show a wide-spread distribution across several continents [26], it is plausible that this organism could also naturally occur in a remote ecosystem such as the Galápagos. Our findings add further support for reptiles as a host of *Ca*. Allocryptoplasma, which had previously only been reported from Europe for the lacertid species *Lacerta viridis* [21], *Lacerta bilineata*, *Podarcis muralis*, and *Podarcis siculus* [13].

On the basis of the detection of 16S rRNA sequences in almost all individuals in our dataset, our results show a high prevalence of *Ca*. Allocryptoplasma among marine iguanas across most of the major islands of the Galápagos archipelago, including San Cristóbal, Santiago, Pinta, Marchena, Genovesa, Isabela, Fernandina, Española, and Floreana. The haplotype network analysis collapsed the marine iguana-derived sequences into a single haplotype, owing to a limitation of the software PopArt, which ignores alignment columns with gaps or ambiguous nucleotides. As a result, the genetic variation within *Ca*. Allocryptoplasma from the Galápagos Islands observed in the ML phylogeny is not represented in the haplotype network. The ambiguous positions observed in the alignments and the topology within the ML phylogeny suggest that multiple strains, or even coinfections of *Ca*. Allocryptoplasma may be present in marine iguanas.

While being relatively common, *Ca*. Allocryptoplasma could have a variable bacterial load between individual marine iguana hosts, as indicated by the high interindividual variability in the number of mapped reads. Only individuals from Isabela consistently exhibited an extremely low number of mapped reads, likely indicating a low bacterial load. Interestingly, this same sampling location on Isabela also had the lowest prevalence for infection with apicomplexan hemoparasites in a different study [33]. As our data are unsuitable for absolute bacterial quantification, this outcome should be investigated in future studies applying appropriate methods such as 16S real-time quantitative polymerase chain reaction (PCR) with specifically designed primers [59].

While the pathogenicity of *Ca*. Allocryptoplasma is currently unknown, it belongs to the family Anaplasmataceae, which includes several animal pathogens [15]. The sister genus of *Ca*. Allocryptoplasma, *Anaplasma*, includes several pathogens [60] responsible for well-known diseases such as human granulocytic anaplasmosis, caused by *Anaplasma phagocytophilum* [61], and canine cyclic thrombocytopenia, caused by *Anaplasma platys* [62]. While *Anaplasma* in vertebrates replicates within membrane-bound vacuoles (morulae) of various types of blood cells [15], details about the replication of *Ca*. Allocryptoplasma in vertebrate and/or invertebrate host cells are currently unknown. In light of the conservation status of marine iguanas as a threatened species [28, 63], it will be important to evaluate the potential impact of *Ca*. Allocryptoplasma on their health. A first step could involve assessing hematocrit levels and analyzing stained blood smears to characterize leukocyte profiles in marine iguanas. Investigating correlations between *Ca*. Allocryptoplasma bacterial load and hematological parameters, body condition, and ectoparasite load could potentially identify pathogenic effects, or help explain the apparently low prevalence observed at the sampling site on Isabela. Stained blood smears might reveal the presence of intracellular inclusions such as morulae. The possibility that marine iguanas may act as a reservoir host for *Ca*. Allocryptoplasma should also be considered, as long-term co-evolution can lead to pathogen tolerance rather than resistance in the host species [64].

*Candidatus* Allocryptoplasma has primarily been identified from ticks [26]. Studies reporting infection in lizards also detected the bacterium in tick species either directly parasitizing lizards or coexisting in the same habitat [13, 21], supporting tick-mediated transmission. Although our study did not assess the prevalence of *Ca*. Allocryptoplasma in any of the four known tick species from the genera *Amblyomma* and *Ornithodoros* that parasitize marine iguanas, it is plausible that these ticks act as reservoirs and vectors of *Ca*. Allocryptoplasma. Besides marine iguanas, these tick species also infest other hosts. *Amblyomma williamsii* and *Amblyomma darwini* parasitize Galápagos land iguanas (*Conolophus subcristatus*), while *A. darwini* has been detected on Galápagos lava lizards (*Microlophus* spp.) [65]. *Ornithodoros darwini* and *Ornithodoros galapagensis* infest two species of Galápagos land iguanas (*Conolophus pallidus* and *C. subcristatus*) and some species of Galápagos lava lizards (*Microlophus* spp.) [31]. *Candidatus* Allocryptoplasma has been detected in several *Amblyomma* ticks, such as *Amblyomma coelebs* [66], *Amblyomma hebraeum* [67], and *Amblyomma tholloni* [26]. This implies that *A. williamsii* and *A. darwini* are prime candidates as potential vectors of *Ca*. Allocryptoplasma.

Marine iguanas are also parasitized by three species of mites in the genus *Vatacarus* [31], found in their nasal fossae. Since *Ca*. Allocryptoplasma has been reported in harvest mites (*Neotrombicula autumnalis*) collected from lacertid lizards [13], transmission by *Vatacarus* mites should be considered. If transmission by any of these acarine parasites is confirmed, it will be important to screen other potential vertebrate hosts, such as *Conolophus* spp. and *Microlophus* spp., to better understand transmission cycles of *Ca*. Allocryptoplasma within the Galápagos ecosystem.

In our 16S rRNA phylogenetic tree, the *A. centrale* sequences were polyphyletic: one grouped with *A. capra* and the other with* A. ovis*. Along with the unresolved placement of the marine iguana-derived sequences within the *Ca*. Allocryptoplasma clade, this suggests that 16S rRNA is inadequate for resolving phylogenetic relationships at lower taxonomic levels [26, 68]. Although this is not the focus of our study, we recommend further investigation of *Ca*. Allocryptoplasma in marine iguanas and other Galápagos taxa using multilocus genotyping with PCR-based sequencing assays. This approach has proven successful for *Ca*. Allocryptoplasma [26], species-level resolution within Anaplasmataceae [67], and within the genus *Anaplasma* [69]. Such assays should also target marker genes such as *groEL*, *rpoB*, *gltA*, and *sucA*, which are single-copy housekeeping genes that are highly informative for resolving species-level phylogenetic relationships in this group [26]. To account for possible coinfections with different strains of the same species, metagenomic methods developed for strain-level resolution could also be applied. Examples include DESMAN [70] or iGDA [71], which leverage long-read sequencing data. On the basis of the unique 16S rRNA haplotype and the phylogenetic placement, our results indicate that the *Ca*. Allocryptoplasma found on Galápagos could represent a separate species within this candidate genus.

## Conclusions

This study highlights the potential of next-generation sequencing for discovering pathogens or endoparasites from host species tissue samples such as blood. RNA sequencing of blood samples from Galápagos marine iguanas revealed a previously unrecognized host association with the putative pathogen *Ca*. Allocryptoplasma, which had not been reported from the Galápagos archipelago before. The results emphasize the need for further research to characterize host–parasite interactions and transmission cycles of both known and putative pathogens affecting marine iguanas, with broader implications for the Galápagos ecosystem.

## Supplementary Information

Additional file 1: Table S1. Mapping success per individual sample, before merging the alignments. Fig. S1. Reads that mapped to the reference 16S ribosomal RNA gene of *Candidatus* Allocryptoplasma sp. Clone LV17, per individual sample, normalized by the respective number of raw rRNA reads and plotted per 100,000. Table S2. List of previously published 16S ribosomal RNA sequences of *Candidatus* Allocryptoplasma that were accessed from GenBank and used in this study. Table S3. List of previously published 16S ribosomal RNA outgroup sequences that were accessed from GenBank and used in this study.

## Data Availability

Newly generated RNA sequencing data have been deposited in the European Nucleotide Archive (ENA) under BioProject no. PRJEB101020. Generated consensus sequences are available on Zenodo under 10.5281/zenodo.17404140. Other data are included in this published article, in its supplementary information files.

## Abbreviations

rRNA: Ribosomal RNA
IUCN: International Union for the Conservation of Nature
mRNA: Messenger RNA
RNA-Seq: RNA sequencing
NCBI: National Center for Biotechnology Information
BLAST: Basic Local Alignment Search Tool
MSA: Multiple sequence alignment
ML: Maximum likelihood
UFboot: Ultrafast bootstrap
SH-aLRT: Shimodaira–Hasegawa-like approximate likelihood ratio test
PCR: Polymerase chain reaction
