# Supplementary material for: Transcriptomic detection of Candidatus Allocryptoplasma (Anaplasmataceae) in Galápagos marine iguanas (Amblyrhynchus cristatus, Iguanidae)
Source: Parasit Vectors. 2025 Nov 28;18:492. doi: 10.1186/s13071-025-07138-7 (PMC12661864; doi:10.1186/s13071-025-07138-7)
Supplement: Supplementary file 1 — Additional file 1: Table S1. Mapping success per individual sample, before merging the alignments. Fig. S1. Reads that mapped to the reference 16S ribosomal RNA gene of Candidatus Allocryptoplasma sp. Clone LV17, per individual sample, normalized by the respective number of raw rRNA reads and plotted per 100,000. Table S2. List of previously published 16S ribosomal RNA sequences of Candidatus Allocryptoplasma that were accessed from GenBank and used in this study. Table S3. List of previously published 16S ribosomal RNA outgroup sequences that were accessed from GenBank and used in this study. [file 13071_2025_7138_MOESM1_ESM.docx]

**Additional file 1: Table S1.** Mapping success per individual sample, before merging the alignments. Shown are the numbers of raw rRNA reads, and the numbers of reads that mapped to the 16S ribosomal RNA gene of *Candidatus* Allocryptoplasma sp. Clone LV17, after removal of sequencing duplicates, before and after normalization by the respective number of raw reads, per 100,000.

| **sample name** | **ENA run accession number** | **sex** | **raw reads** | **mapped reads** | **normalized mapped reads per 100,000** |
| --- | --- | --- | --- | --- | --- |
| ESP16-241 | ERR15747456 | male | 931221 | 1733 | 186.10 |
| ESP16-244 | ERR15747457 | male | 763929 | 304 | 39.79 |
| ESP16-245 | ERR15747458 | female | 583716 | 117 | 20.04 |
| ESP16-246 | ERR15747459 | female | 1081002 | 282 | 26.09 |
| ESP16-247 | ERR15747460 | female | 975274 | 1888 | 193.59 |
| ESP16-250 | ERR15747461 | male | 816178 | 334 | 40.92 |
| FDA16-331 | ERR15747462 | male | 626146 | 28 | 4.47 |
| FDA16-332 | ERR15747463 | female | 736546 | 26 | 3.53 |
| FDA16-333 | ERR15747464 | male | 1559999 | 8 | 0.51 |
| FDA16-334 | ERR15747465 | male | 1037070 | 3 | 0.29 |
| FDA16-335 | ERR15747466 | male | 889416 | 2 | 0.22 |
| FDA16-336 | ERR15747653 | male | 673564 | 41 | 6.09 |
| FDA16-337 | ERR15747654 | female | 333155 | 278 | 83.44 |
| FL16-274 | ERR15747655 | male | 735045 | 19 | 2.58 |
| FL16-280 | ERR15747656 | male | 1050089 | 37 | 3.52 |
| FL16-288 | ERR15747657 | male | 781972 | 118 | 15.09 |
| FL16-289 | ERR15747658 | female | 1044526 | 181 | 17.33 |
| FL16-293 | ERR15747659 | female | 1319338 | 1419 | 107.55 |
| FL16-300 | ERR15747660 | male | 621993 | 17 | 2.73 |
| GEN16-10 | ERR15747668 | male | 504561 | 2 | 0.40 |
| GEN16-12 | ERR15747669 | male | 710969 | 159 | 22.36 |
| GEN16-2 | ERR15747670 | male | 747634 | 24 | 3.21 |
| GEN16-5 | ERR15747671 | male | 594295 | 242 | 40.72 |
| GEN16-7 | ERR15747672 | male | 1053997 | 188 | 17.84 |
| GEN16-8 | ERR15747673 | male | 999518 | 376 | 37.62 |
| IS16-371 | ERR15747674 | male | 909838 | 2 | 0.22 |
| IS16-374 | ERR15747675 | male | 391689 | 4 | 1.02 |
| IS16-383 | ERR15747676 | male | 643086 | 4 | 0.62 |
| IS16-384 | ERR15747677 | male | 603641 | 12 | 1.99 |
| IS16-386 | ERR15747678 | male | 750759 | 4 | 0.53 |
| IS16-389 | ERR15747679 | male | 727169 | 10 | 1.38 |
| MAR16-31 | ERR15747680 | male | 574335 | 51 | 8.88 |
| MAR16-32 | ERR15747681 | male | 697345 | 106 | 15.20 |
| MAR16-33 | ERR15747682 | male | 574448 | 11 | 1.91 |
| MAR16-39 | ERR15747683 | female | 768997 | 57 | 7.41 |
| MAR16-45 | ERR15747684 | male | 447236 | 0 | 0.00 |
| MAR16-46 | ERR15747685 | female | 683067 | 148 | 21.67 |
| PIN16-61 | ERR15747687 | male | 793747 | 607 | 76.47 |
| PIN16-62 | ERR15747688 | female | 697262 | 30 | 4.30 |
| PIN16-64 | ERR15747689 | male | 414829 | 157 | 37.85 |
| PIN16-65 | ERR15747690 | male | 750686 | 418 | 55.68 |
| PIN16-66 | ERR15747697 | female | 3870536 | 1441 | 37.23 |
| PIN16-67 | ERR15747698 | female | 748216 | 2004 | 267.84 |
| SAN16-100 | ERR15747700 | female | 706040 | 111 | 15.72 |
| SAN16-93 | ERR15747701 | male | 414225 | 84 | 20.28 |
| SAN16-94 | ERR15747702 | male | 565931 | 64 | 11.31 |
| SAN16-95 | ERR15747703 | male | 783916 | 128 | 16.33 |
| SAN16-96 | ERR15747704 | female | 508038 | 79 | 15.55 |
| SAN16-97 | ERR15747705 | male | 681676 | 504 | 73.94 |
| SAN16-98 | ERR15747706 | male | 700787 | 48 | 6.85 |
| SRL16-153 | ERR15747707 | male | 665631 | 17 | 2.55 |
| SRL16-154 | ERR15747708 | female | 1390579 | 753 | 54.15 |
| SRL16-155 | ERR15747709 | female | 1223623 | 1313 | 107.30 |
| SRL16-156 | ERR15747710 | male | 1078274 | 22 | 2.04 |
| SRL16-157 | ERR15747711 | male | 892805 | 40 | 4.48 |
| SRL16-158 | ERR15747712 | female | 1175663 | 948 | 80.64 |

**Additional file 1: Fig. S1.** Reads that mapped to the reference 16S ribosomal RNA gene of *Candidatus* Allocryptoplasma sp. Clone LV17, per individual sample, normalized by the respective number of raw rRNA reads and plotted per 100,000.


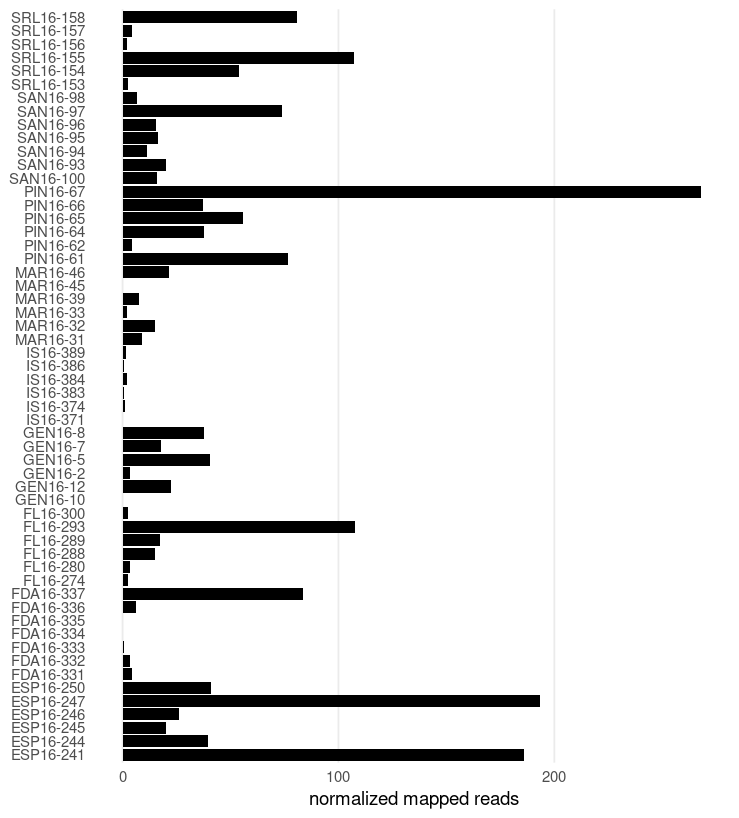


**Additional file 1: Table S2.** List of publicly available 16S ribosomal RNA sequences of *Candidatus* Allocryptoplasma that were accessed from GenBank and used in this study.

| **Genbank Accession Nr.** | **Name** | **Host [Country]** | **Superscript Number in Fig. 1-2** |
| --- | --- | --- | --- |
| KP276586.1 | *Candidatus* Allocryptoplasma californiense isolate CC-14 | *Ixodes pacificus* [California, USA] | 1 |
| KP276587.1 | *Candidatus* Allocryptoplasma californiense isolate CP-1 | *Ixodes pacificus*  [California, USA] | 1 |
| KP276585.1 | *Candidatus* Allocryptoplasma californiense isolate MR-9 | *Ixodes pacificus*  [California, USA] | 1 |
| OQ724852.1 | *Candidatus* Allocryptoplasma sp. isolate Irici10 | *Ixodes ricinus*  [France] | 2 |
| OQ724844.1 | *Candidatus* Allocryptoplasma sp. isolate Irici2 | *Ixodes ricinus*  [France] | 2 |
| OQ724850.1 | *Candidatus* Allocryptoplasma sp. isolate Irici8 | *Ixodes ricinus*  [France] | 2 |
| OQ724843.1 | *Candidatus* Allocryptoplasma sp. isolate Irici1 | *Ixodes ricinus*  [France] | 2 |
| OQ724847.1 | *Candidatus* Allocryptoplasma sp. isolate Irici5 | *Ixodes ricinus*  [France] | 2 |
| OQ724848.1 | *Candidatus* Allocryptoplasma sp. isolate Irici6 | *Ixodes ricinus*  [France] | 2 |
| OQ724849.1 | *Candidatus* Allocryptoplasma sp. isolate Irici7 | *Ixodes ricinus*  [France] | 2 |
| OQ724845.1 | *Candidatus* Allocryptoplasma sp. isolate Irici3 | *Ixodes ricinus*  [France] | 2 |
| OQ724846.1 | *Candidatus* Allocryptoplasma sp. isolate Irici4 | *Ixodes ricinus*  [France] | 2 |
| OQ724851.1 | *Candidatus* Allocryptoplasma sp. isolate Irici9 | *Ixodes ricinus*  [France] | 2 |
| MG924904.1 | *Candidatus* Allocryptoplasma sp. clone LV17 | *Lacerta viridis*  [Slovakia] | 3 |
| OQ724839.1 | *Candidatus* Allocryptoplasma sp. isolate AcN0702 | *Amblyomma coelebs*  [French Guiana] | 4 |
| OQ724842.1 | *Candidatus* Allocryptoplasma sp. isolate 82D | *Haemaphysalis parmata* [Uganda] | 5 |
| OQ724840.1 | *Candidatus* Allocryptoplasma sp. isolate 82A | *Haemaphysalis parmata*  [Uganda] | 5 |
| OQ724841.1 | *Candidatus* Allocryptoplasma sp. isolate 82C | *Haemaphysalis parmata* [Uganda] | 5 |
| OQ724862.1 | *Candidatus* Allocryptoplasma sp. isolate AthollJ | *Amblyomma tholloni*  [Uganda] | 8 |
| OQ724857.1 | *Candidatus* Allocryptoplasma sp. isolate AthollE | *Amblyomma tholloni*  [Uganda] | 8 |
| OQ724858.1 | *Candidatus* Allocryptoplasma sp. isolate AthollF | *Amblyomma tholloni*  [Uganda] | 8 |
| OQ724855.1 | *Candidatus* Allocryptoplasma sp. isolate AthollC | *Amblyomma tholloni*  [Uganda] | 8 |
| OQ724859.1 | *Candidatus* Allocryptoplasma sp. isolate AthollG | *Amblyomma tholloni*  [Uganda] | 8 |
| OQ724853.1 | *Candidatus* Allocryptoplasma sp. isolate AthollA | *Amblyomma tholloni*  [Uganda] | 8 |
| OQ724860.1 | *Candidatus* Allocryptoplasma sp. isolate AthollH | *Amblyomma tholloni*  [Uganda] | 8 |
| OQ724856.1 | *Candidatus* Allocryptoplasma sp. isolate AthollD | *Amblyomma tholloni*  [Uganda] | 8 |
| OQ724854.1 | *Candidatus* Allocryptoplasma sp. isolate AthollB | *Amblyomma tholloni*  [Uganda] | 8 |
| OQ724861.1 | *Candidatus* Allocryptoplasma sp. isolate AthollI | *Amblyomma tholloni*  [Uganda] | 8 |
| GU075700.1 | *Candidatus* Allocryptoplasma clone HLAE107 | *Haemaphysalis longicornis*  [South Korea] | 6 |
| GU075702.1 | *Candidatus* Allocryptoplasma clone HLAE444 | *Haemaphysalis longicornis*  [South Korea] | 6 |
| GU075703.1 | *Candidatus* Allocryptoplasma clone HLAE333 | *Haemaphysalis longicornis*  [South Korea] | 6 |
| GU075704.1 | *Candidatus* Allocryptoplasma clone HLAE344 | *Haemaphysalis longicornis*  [South Korea] | 6 |
| JN715833.1 | *Candidatus* Allocryptoplasma clone BJ01 | *Haemaphysalis longicornis*  [China] | 6 |
| GU075701.1 | Uncultured *Anaplasma* sp. clone HLAE431 16S ribosomal RNA gene, partial sequence | *Haemaphysalis longicornis*  [South Korea] | 6 |
| GU075699.1 | Uncultured *Anaplasma* sp. clone HLAE143 16S ribosomal RNA gene, partial sequence | *Haemaphysalis longicornis*  [South Korea] | 6 |
| OP480171.1 | Uncultured *Anaplasma* sp. clone Ac152 16S ribosomal RNA gene, partial sequence | *Amblyomma calcaratum*  [Argentina] | 7 |
| OQ092427.1 | Uncultured *Anaplasma* sp. clone AnaAt1 16S ribosomal RNA gene, partial sequence | *Amblyomma tholloni*  [Uganda] | 8 |
| OQ092428.1 | Uncultured Anaplasma sp. clone AnaHp1 16S ribosomal RNA gene, partial sequence | *Haemaphysalis parmata*  [Uganda] | 5 |

**Additional file 1: Table S3.** List of publicly available 16S ribosomal RNA outgroup sequences that were accessed from GenBank and used in this study**.**

| **Genbank Accession Nr.** | **Name** | **Superscript Number in Fig. 1** |
| --- | --- | --- |
| CP015994.2 | *Anaplasma ovis* str. Haibei | 10 |
| AF309865.1 | *Anaplasma ovis* strain Idaho | 9 |
| CP001079.1 | *Anaplasma marginale* str. Florida | 12 |
| KT264188.1 | *Anaplasma marginale* isolate AmCU01 | 13 |
| CP000235.1 | *Anaplasma phagocytophilum* HZ | 22 |
| CP035303.1 | *Anaplasma phagocytophilum* strain KZ-A1 | 23 |
| NZ_CP046391.1 | *Anaplasma platys* strain S3 | 20 |
| EF139459.1 | *Anaplasma platys* | 21 |
| CP001759.1 | *Anaplasma centrale* str. Israel | 11 |
| AF283007.1 | *Anaplasma centrale* | 15 |
| LC432126.1 | *Anaplasma capra* KWD-35 | 16 |
| MW721591.1 | *Anaplasma capra* strain Hstaji200 | 14 |
| MH255936.1 | *Anaplasma bovis* isolate Erlicun-cattle-18 | 17 |
| KY425447.1 | *Anaplasma bovis* isolate Y258 | 18 |
| NR_118489.1 | *Anaplasma odocoilei* strain UMUM76 | 19 |
| CP089285.1 | *Neoehrlichia mikurensis* isolate 18-2804 | 24 |
| EF633744.1 | *Candidatus* Neoehrlichia lotoris RAC413 | 25 |
| CP040117.1 | *Ehrlichia ruminantium* strain Nonile | 26 |
| CP006917.1 | *Ehrlichia muris* AS145 | 27 |
| CP041924.1 | *Wolbachia pipientis* strain wAlbB-HN2016 | 28 |
| NZ_HG810405.1 | *Wolbachia* endosymbiont of *Onchocerca volvulus* str Cameroon chromosome I | 29 |
| NR_074389.1 | *Neorickettsia* *risticii* str. Illinois | 30 |
| CP000237.1 | *Neorickettsia sennetsu* strain Miyayama | 31 |
| NR_118776.1 | *Rickettsia parkeri* strain Maculatum-20 | 32 |
